# Implementation of multiple-instance learning in drug activity prediction

**DOI:** 10.1186/1471-2105-13-S15-S3

**Published:** 2012-09-11

**Authors:** Gang Fu, Xiaofei Nan, Haining Liu, Ronak Y Patel, Pankaj R Daga, Yixin Chen, Dawn E Wilkins, Robert J Doerksen

**Affiliations:** 1 Department of Medicinal Chemistry, School of Pharmacy, University of Mississippi, University, 38677, USA; 2 Department of Computer and Information Science, School of Engineering, University of Mississippi, University, 38677, USA; 3 Research Institute of Pharmaceutical Sciences, School of Pharmacy, University of Mississippi, University, 38677, USA; 4 Department of Genetics, Washington University School of Medicine, 4444 Forest Park Blvd., Campus Box 8510, MO 63108, USA

## Abstract

**Background:**

In the context of drug discovery and development, much effort has been exerted to determine which conformers of a given molecule are responsible for the observed biological activity. In this work we aimed to predict bioactive conformers using a variant of supervised learning, named multiple-instance learning. A single molecule, treated as a bag of conformers, is biologically active if and only if at least one of its conformers, treated as an instance, is responsible for the observed bioactivity; and a molecule is inactive if none of its conformers is responsible for the observed bioactivity. The implementation requires instance-based embedding, and joint feature selection and classification. The goal of the present project is to implement multiple-instance learning in drug activity prediction, and subsequently to identify the bioactive conformers for each molecule.

**Methods:**

We encoded the 3-dimensional structures using pharmacophore fingerprints which are binary strings, and accomplished instance-based embedding using calculated dissimilarity distances. Four dissimilarity measures were employed and their performances were compared. 1-norm SVM was used for joint feature selection and classification. The approach was applied to four data sets, and the best proposed model for each data set was determined by using the dissimilarity measure yielding the smallest number of selected features.

**Results:**

The predictive abilities of the proposed approach were compared with three classical predictive models without instance-based embedding. The proposed approach produced the best predictive models for one data set and second best predictive models for the rest of the data sets, based on the external validations. To validate the ability of the proposed approach to find bioactive conformers, 12 small molecules with co-crystallized structures were seeded in one data set. 10 out of 12 co-crystallized structures were indeed identified as significant conformers using the proposed approach.

**Conclusions:**

The proposed approach was proven not to suffer from overfitting and to be highly competitive with classical predictive models, so it is very powerful for drug activity prediction. The approach was also validated as a useful method for pursuit of bioactive conformers.

## Background

In the context of drug discovery research, it is challenging but of great importance to be able to determine which 3-dimensional (3D) shapes (so-called conformers) of a given molecule are responsible for its observed biological activity. Due to structural flexibility, a molecule may adopt a wide range of conformers and the identification of the bioactive conformers is extremely important in order to understand the recognition mechanism between small molecules and proteins, which is crucial in drug discovery and development. Until now, the most reliable approach to obtain the bioactive conformer is to use the X-ray crystal structure of a ligand-protein complex; however, the number of such structures is limited because of the experimental difficulty in obtaining the crystals, especially for transmembrane proteins, such as G protein-coupled receptors (GPCR) [1,2] and membrane transporters. We were interested to apply to this problem a machine-learning approach which does not require crystal structures, named multiple-instance learning (MIL) via embedded instance selection (MILES). MILES has been demonstrated as an efficient and accurate approach to solve different multiple-instance problems [3], in particular, to predict drug activity using Musk data sets. In the context of drug activity prediction, MILES enables the construction of a quantitative structure-activity relationship (QSAR) model, and subsequently the identification of bioactive conformers.

**Figure 1:**
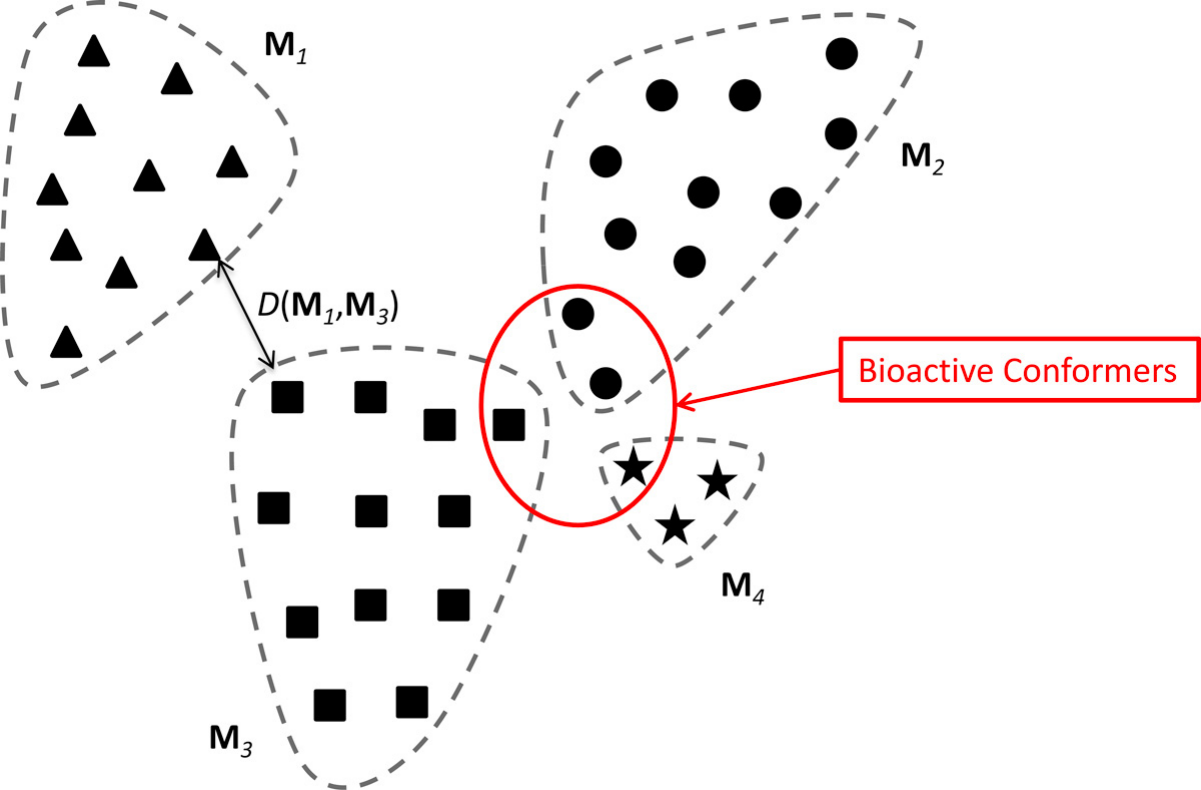
**Cartoon representation of the relationship between molecules and conformers**. **M***_i_, i *= 1, 2, 3, 4 represent the molecules (bags), circled by dashed lines. The solid triangles in **M***_1_*, circles in **M***_2_*, squares in **M***_3_*, and stars in **M***_4 _*represent conformers for different molecules. Molecules *2, 3*, and *4 *were biologically active since they had at least one bioactive conformer, whereas molecule *1 *was inactive since none of its conformers was bioactive. The distance between two molecules, **M***_1 _*and **M***_3_*, was calculated by the minimum distance *D*(**M***_1_*, **M***_3_*).

MIL is a variant of supervised learning, and it has been applied for a variety of learning problems including drug activity prediction [4], image database retrieval [5], text categorization [6], and natural scene classification [7]. In the context of drug activity prediction, the observed biological activity is associated with a single molecule (bag) without knowing which conformer or conformers (instances) are responsible. Furthermore, a molecule is biologically active if and only if at least one of its conformers is responsible for the observed bioactivity; and the molecule is inactive if none of its conformers is responsible (Figure 1). A difficulty in implementation arises from the fact that different molecules have a different number of conformers, since some molecules having multiple rotatable bonds are highly flexible and others with rigid structures only have a small numbers of conformers.

**Figure 2:**
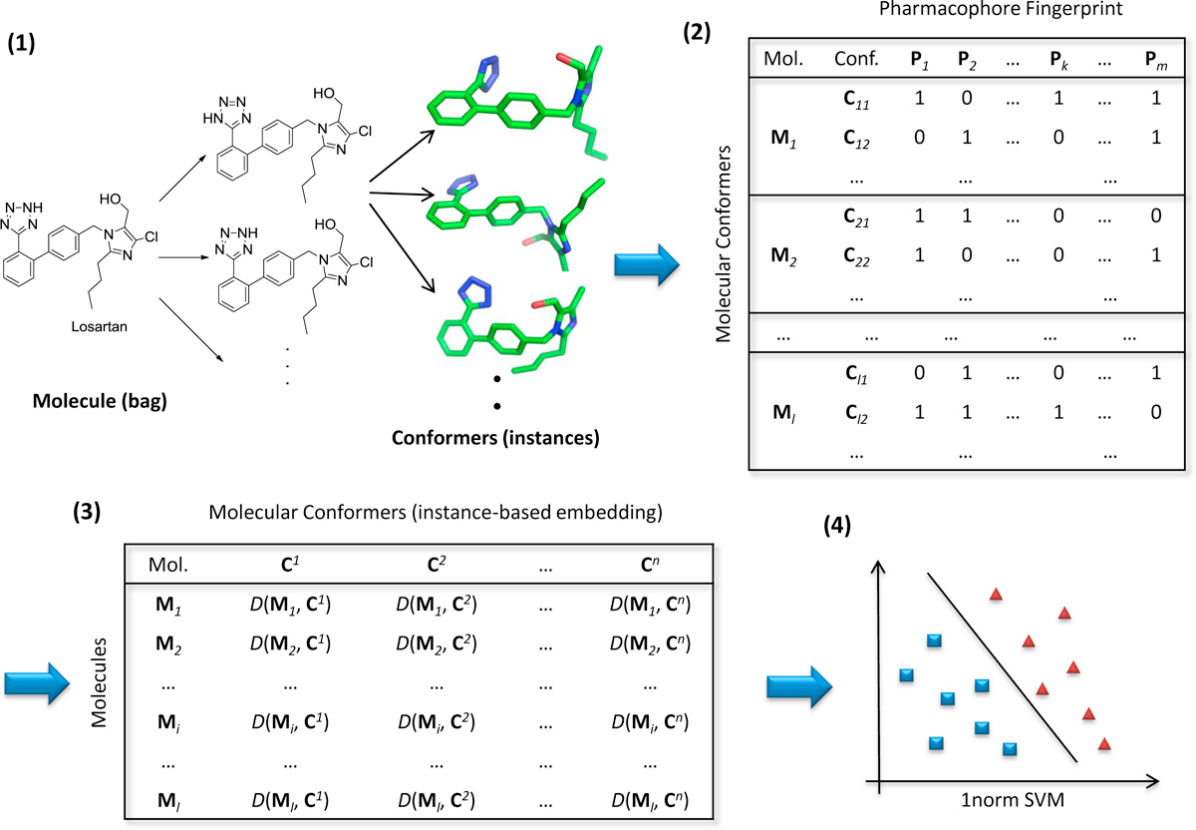
**Overview of the MILES approach**. (1) Structure preprocessing and conformational sampling. (2) Creating pharmacophore fingerprints and significance analysis of pharmacophore models. (3) Instance-based feature mapping based on structural similarity measures. (4) Joint feature selection and classification using 1-norm SVM.

The overall strategy for structural and data mining using MILES (Figure 2) is summarized here. First of all, a complete sampling of conformational space provides a large number of conformers for each molecule. The molecules are themselves each already labelled as either positive or negative. However, the labels for the conformers are unavailable during the model generation. Each conformer is denoted by a unique pharmacophore fingerprint which is a superior feature-based 3D descriptor unveiling structural similarity and diversity [8-11]. The pharmacophore fingerprint is encoded into a binary string which indicates the presence or absence of a match to individual pharmacophore models. Since the exhaustively enumerated fingerprints have millions of bits, which may be beyond computational limits, a significance analysis of pharmacophore models [12] is employed to determine the optimal subset of bits of the fingerprint. Subsequently, MILES converts the MIL to a standard supervised learning problem by embedding bags (molecules) into an instance-based (conformer-based) feature space via structural dissimilarity measures [13]. Finally, 1-norm SVM is applied to select the most important features, identifying the highly significant conformers which help the most to distinguish active and inactive molecules, and, simultaneously, to construct a predictive classification model.

In the present work, MILES has been applied to study the biological activities of several sets of molecules interacting with different receptor targets including glycogen synthase kinase-3 (GSK-3), cannabinoid receptors (CBrs), and P-glycoprotein (P-gp). All of these receptors have been emerging as increasingly important therapeutic targets. GSK-3 is a multifunctional serine/threonine protein kinase involved in the regulation of a wide range of cellular functions, including glucose metabolism, neuronal processes, chronic inflammation, cell proliferation and apoptosis [14]. CBrs are a class of GPCRs and have been targeted for various disease conditions such as obesity, drug abuse disorders, inflammatory diseases, anorexia and vomiting [15]. P-gp, a membrane transporter, is responsible for drug efflux and multidrug resistance, especially to cancer drugs [16]. Except for GSK-3, the other proteins are membrane-associated and there is no available crystal structure for them. The identification of bioactive conformers for the molecules targeting membrane-associated proteins using MILES could be highly informative and desirable. Identified conformers can be used in various drug discovery approaches such as scaffold hopping, target fishing, and 3D structural alignment for 3D quantitative structure-activity relationship (QSAR) studies.

Based on our calculations, MILES is highly competitive with the classical QSAR approaches which do not include instance-based feature mapping in terms of predictive abilities. Meanwhile, we have validated that MILES has the ability to identify a subset of highly relevant conformers, including the bioactive conformers, which contribute to the classification of active and inactive molecules.

## Methods

### Data set preparation

Four different data sets were compiled through extensive literature search. Data set **I **includes all molecules exhibiting inhibitory activities for human GSK-3. Data sets **II **and **III **contain molecules modulating the intracellular activities of human CBrs. Since there are two identified CBr subtypes, CB1 and CB2, two different data sets were prepared to study the protein-small molecule interactions of the receptors separately. Some of the molecules which have reported binding affinities for both CB subtypes were included in both data sets **II **and **III**. Data set **IV **contained compounds which had been tested as substrates of P-gp.

The molecules collected for each data set were labelled as either positive or negative. A positive molecule has a high binding affinity with the target protein, whereas a negative molecule has a low binding affinity. A single cutoff value has been widely used in the development of classification models. However, it is inaccurate to use a single cutoff value for the separation of continuous biological activities in the context of drug activity prediction. The biological activities are represented by continuous numbers, and the small differences between the values above and below the cutoff value cannot imply the distinct nature of binding affinity. Furthermore, the small difference in the bioassay results may arise from systematic errors introduced by different experimental protocols used in different labs, so it cannot be used as solid evidence for the classification of molecules. Therefore, multiple cutoff values were employed to separate molecules into positive and negative classes.

For data set **I**, the molecules were categorized into positive and negative molecules using cutoff values of *IC*_50 _≤ 50 nM and *IC*_50 _≥ 500 nM, respectively. The molecules having inhibitory activities between the two cutoff values were considered as moderately active molecules, and were discarded from the data set. The wide margin between the two cutoff values was used to account for the variances in biological assays. For data set **II **and **III**, the molecules were classified as positive if the *K*_i _≤ 50 nM or *IC*_50 _≤ 100 nM or *EC*_50 _≤ 100 nM (*IC*_50 _is approximately twice as large as *K*_i _based on the definition); and the molecules were classified as negative if the *K*_i _≥ 500 nM or *IC*_50 _≥ 1000 nM or *EC*_50 _≥ 1000 nM. The labels for the molecules in data set **IV **indicated whether or not the molecule is a substrate for the target protein. They were obtained from the literature [12].

### Division of training and test set

External validation was achieved using an independent test set. The split of the data set into training and test sets was carried out using Kohonen self-organizing maps (SOM) in Canvas 1.4 from Schrödinger Suite 2011. The SOM is trained using unsupervised learning to produce a square 2D grid map from the high dimensional input space. Each grid cell (neuron) contains a cluster of structurally similar molecules defined by the input vectors. The SOM takes advantage of clustering capabilities so that the selected training set can represent the independent test set in terms of the input space and chemical domains. Molecular pharmacophore fingerprints were used to describe the relevant structural information of the molecules and were used as input variables to build the SOM. The grid size of the map depends on the number of molecules in the data set. For data sets **I, II**, and **III**, the Kohonen maps built included 10 × 10 neurons and 500 epochs. For the data set **IV**, a Kohonen map consisting of 8 × 8 neurons and 500 epochs was built. The molecules were then stratified and sampled from each neuron to select the training and test set molecules.

### Preprocessing and conformational sampling

The molecules (bags) can be represented by **M***_i_, i *= 1, ⋯, *l *where *l *is the total number of molecules. The 3D molecular structures were generated using the Ligprep module from Schrödinger Suite 2011, and then subjected to preprocessing to enumerate all the possible tautomers. The protonation states of ionizable groups were set to match pH = 7.4, and the stereochemistry was retained from the original 3D structures. In order to explore the conformational space exhaustively, the mixed torsional/low mode sampling method was employed, using MacroModel from Schrödinger Suite 2011. The torsional sampling involves multiple Monte Carlo minimum searches for global exploration, and the low mode conformational search allows for automatic local exploration. The torsional increment for each rotatable bond was set to 15° and the maximum number of total steps for torsional sampling was 1,000. The energy window for saving structures was set to 83.7 kJ/mol (20 kcal/mol). The small torsional increment and wide energy window were employed to provide a reasonable coverage of the conformational space. Each enumerated conformer was energy minimized to eliminate unreasonable geometries and reduce internal steric clashes, using the Polak-Ribière conjugate gradient method with a gradient convergence threshold of 0.05 and a maximum of 500 iterations. To remove redundant conformations, the maximum atom deviation cutoff was set to 1.5 Å. As a result, each molecule **M***_i _*has several possible conformers **C***_ij_, j *= 1, ⋯, *n_i_*, where *n_i _*is the number of conformers (instances) for molecule *i*.

In order to validate that MILES can identify the bioactive conformers, we seeded 12 co-crystallized conformers, one for each of 12 molecules, in the set of sampled conformers for data set **I**. The validation process will be described in the following sections.

### Generation of pharmacophore fingerprints

The pharmacophore fingerprint as a measure of molecular similarity and diversity based on 3D pharmacophoric shape was enumerated using Canvas 1.4 from Schrödinger Suite 2011. Each pharmacophore fingerprint associated with a unique conformer can be represented by a binary string, such as **P***_ij _*= {**p***_1_*, ⋯, **p***_k_*, ⋯, **p***_m_*} and encodes quantitative structural information for conformer **C***_ij_*, where each bit value **p***_k_, k *= 1, ⋯, *m *indicates the presence or absence of a match to a single pharmacophore model, representing a unique 3D arrangement of a number of pharmacophore features. If the conformer fits the pharmacophore model for a particular *k*, in other words if the functional groups of the conformer fully overlap on all the pharmacophore features in the model, **p***_k _*equals 1; otherwise, **p***_k _*equals 0. As a result, each conformer is associated with a unique pharmacophore fingerprint as a conformational signature, which enables us to describe quantitatively the 3D structural information. The pharmacophore features employed in the models consist of hydrogen bond donor (D), hydrogen bond acceptor (A), hydrophobic group (H), negatively charged group (N), positively charged group (P), and aromatic ring (A). In the present study, only four-feature based models were employed in order to allow a reasonable description of 3D orientation of the structures and retain information about molecular chirality, which is lost in three-feature based models. Different combinations of four out of six pharmacophore features were exhaustively enumerated and inter-feature distances were varied from 2.0 Å to 20.0 Å to form the different pharmacophore models. Each pharmacophore feature was treated as a bin with width 2.0 Å, and the bin overlap threshold was 1.0 Å. To fit to a model the conformer must fit to each of the four features in the model. The maximum distance between pharmacophore features was set to 20.0 Å in order to be able to cover the largest molecular structures in the databases. The originally enumerated fingerprints were subject to occurrence-based filtering to remove the pharmacophore models present in less than 5% of the total number of molecules, since the pharmacophore models with a very low occurrence are not useful for discriminating between positive and negative classes.

### Significance analysis of pharmacophore models

The post-filtered pharmacophore fingerprints still have too many bits that lack information content, as indicated by too many '0' values. Therefore a nonparametric supervised learning approach, motivated by the significance analysis of microarrays (SAM) algorithm proposed by Tibshirani *et al. *[17], was applied to elucidate a consistent pattern from the numerous bits of pharmacophore fingerprints. The detailed implementation and customization of the relevant procedures has been described in [12]. The ranking score for each pharmacophore model was computed based on a two-class *t*-statistic, which calculates the ratio of the difference of occurrences of that model in positive and negative classes and compares to the standard deviation of occurrence measures. Pharmacophore models with ranking scores greater than a threshold have statistical significance, where the threshold was computed at the 90th percentile among 500 random permutations of the class labels across all the molecules. In order to distinguish truly significant and falsely significant pharmacophore models, that ranking score serving as a true score was then compared with a reference score computed from the same set of random permutations. If the difference between the true score and the reference score exceeds a cutoff threshold (called Δ) then the pharmacophore model is truly significant; otherwise it is falsely significant.

### Instance-based feature mapping

MILES provides a framework to convert a MIL problem to a standard supervised learning problem via instance-based embedding. All the conformers (instances) belong to the instance-based feature space. For convenience, all conformers in all molecules were lined up together, and were re-indexed in the embedded feature space as **C***^r^, r *= 1, ⋯, *n *where $n=\sum_{i=1}^{l}n_{i}$. Instance-based feature mapping can be accomplished using calculated structural dissimilarities. Different binary string distance measures were tested, including the Soergel distance, Dice distance, Manhattan distance, and Rogers-Tanimoto distance (Table 1). The range of each dissimilarity measure was normalized to be [0, 1] by definition. Given a conformer **C***_ij _*denoted by a binary string **P***_ij_*, the dissimilarity measure, denoted as *D*(**C***_ij_*, **C***^r^*), is calculated based on the number of occurrences of bit matches. Since one molecule is defined as a bag of multiple conformers (instances), the dissimilarity measure for a molecule, denoted as *D*(**M***_i_*, **C***^r^*), is calculated based on the minimum distance using the closest instance in the bag for **M***_i_*:

**Table 1:** Metrics used for dissimilarity measurements

Dissimilarity Measure	**Definition***^**a**^*
Soergel	$\frac{b+c}{a+b+c}$
Dice	$\frac{b+c}{2a+b+c}$
Manhattan	$\frac{b+c}{a+b+c+d}$
Rogers-Tanimoto	$\frac{2\times \left(b+c\right)}{a+d+2\times \left(b+c\right)}$

*^a ^*Let P1 and P2 be two pharmacophore fingerprints, *a *be the count of bits which are set to 1 in both P1 and P2, *b *be the count of bits which are set to 1 in P1 but not in P2, *c *be the count of bits which are set to 1 in P2 but not in P1, and *d *be the count of bits which are set to 0 in both P1 and P2. So *a *is called the number of total matches, *b *and *c *are called the number of single matches, and *d *is called the number of no matches.

(1) $$D\left(M_{i},C^{r}\right)=\underset{j}{\text{min}}\;D\left(C_{ij},C^{r}\right)$$

The minimum distance calculation (Figure 1) extends the idea of the diverse density framework proposed for instance-based learning [18].

### Joint feature selection and classification

Since the molecules in the training sets are highly flexible, instance-based embedding, which provides a framework to convert a MIL problem to a traditional supervised learning problem, may produce a very high dimensional feature space. But many features are redundant or irrelevant, and do not play an important role in the classification of molecules as positive or negative. So an efficient feature selection model is required for selection of an optimal subset of instance-based features. Considering its excellent performance in many applications [19], the 1-norm SVM method was chosen as a joint approach to construct classifiers and to select important features simultaneously. The prediction model can be formulated as a linear classifier,

(2) $$y=sign\left(\omega^{T}m+b\right)$$

where *y *denotes the class label as either positive or negative; *ω *and *b *are model parameters which are optimized during model generation; and ***m ***corresponds to a molecule (bag), which is defined by an *n*-dimensional vector of dissimilarities calculated using (1), i.e., dissimilarities with respect to all conformers in all molecules. The domain to (2) is therefore the space of **R***^n^*, where *n *is the sum of all conformers in all molecules. The SVM approach constructs classifiers based on hyperplanes by minimizing a regularized training error, *ξ*_training_,

(3) $$\lambda P\left[•\right]+\xi_{\text{training}}$$

where *P*[•] is a regularizer, and *λ *is the regularization parameter, the only tuning parameter to be optimized by the user. In 1-norm SVM, the regularizer is chosen to be the 1-norm of the weight vector,

(4) $${∥\omega ∥}_{1}=\sum_{r}\left|\omega_{r}\right|.$$

1-norm regularization favors sparse solutions, i.e., it drives many components of *ω *to zero.

Once the optimal solution, with values ω* and b*, is obtained, the magnitude of its component $\omega_{r}^{*}$ indicates the significance of the *r*-th feature (conformer) in the instance-based feature space. The features corresponding to non-zero entries in ω* are selected as important features, which are given as a set $\Gamma =\left\{r:\left|\omega_{r}^{*}\right|>0\right\}$). They are needed for the classification problem of interest

(5) $$y=sign\left(\sum_{r\in \Gamma }\omega_{r}^{*}D\left(M_{i},C^{r}\right)+b^{*}\right).$$

Note that (2) is equivalent to (5) where all weights with 0 values are ignored. The domain of (5) is **R***^|Γ|^*, a subspace of **R***^n^*, defined by conformers whose weights are nonzero. The features selected as important are called *prototype conformers*. The plus or minus sign of $\omega_{r}^{*}$ indicates the positive or negative contribution, respectively, of the *r*-th prototype conformer to the putative bioactive conformers for each individual molecule.

Our formulation of MILES works directly on a dissimilarity mapping, which is different from a similarity mapping described by Chen, *et al. *[3]. One can transform a dissimilarity mapping to a similarity mapping via an exponential function. However, this would introduce an additional super parameter, σ. Although, a proper choice of σ could improve the performance of a model, the selection of a proper value for σ increases the computational cost significantly. Hence we use a dissimilarity mapping to reduce the computational cost.

### Identification of bioactive conformers

One appealing advantage of the MILES algorithm is that it can identify the most significant instances in a bag according to their contributions to the classification of that bag. In the context of drug activity prediction, we can identify the most significant conformers, called the bioactive conformers, for each molecule. The putative bioactive conformers are the conformers that contributed the most to the classification of positive and negative molecules.

**Figure 3:**
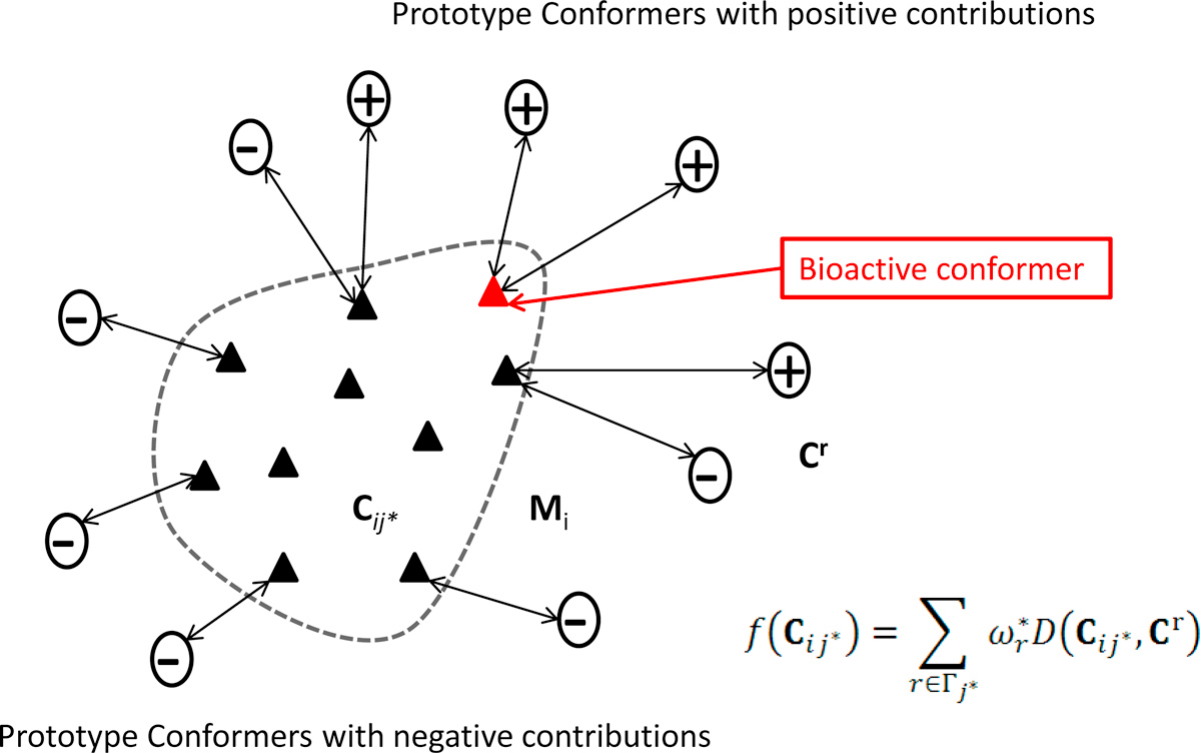
**Identification of bioactive conformers**. Molecule *i *was circled by a dashed line and its conformers were represented by solid triangles. The plus circles represent the positively contributing prototype conformers and the minus circles represent the negatively contributing prototype conformers. The identification of bioactive conformers was accomplished by calculating the total contributions from the closest prototype conformers.

The identification of bioactive conformers can be accomplished with the assistance of the prototype conformers mentioned above (Figure 3). Given a molecule **M***_i _*with its conformers **C***_ij_, j *= 1, ⋯, *n_i_*, we define an index set ∑ = {j*: j* = argmin*_j _D*(**C***_ij_*, **C***^r^*), *r *∈ Γ}), which includes the index for conformers closest to each prototype conformer. Hence, Σ defines a minimal set of conformers, called *significant conformers*, which are responsible for the classification of **M***_i_*. By definition, each prototype conformer in set Γ has a single conformer in set Σ closest to it, but each significant conformer in set Σ may have multiple prototype conformers in set Γ closest to it. So we need to define an index set for each significant conformer in set Σ that includes the index for the prototype conformers closest to it, which is given as $\Gamma_{j^{*}}=\left\{r:r\in \Gamma ,j^{*}=\text{arg}{\text{min}}_{j}D\left(C_{ij},C^{r}\right)\right\}$). As a result, the contribution of each significant conformer to the classification of molecule can be calculated as

(6) $$f\left(C_{ij^{*}}\right)=\underset{r\in \Gamma_{j^{*}}}{\sum }\omega_{r}^{*}D\left(C_{ij^{*}},C^{r}\right)$$

where$f\left(C_{ij^{*}}\right)$ denotes the contribution of the conformer $C_{ij^{*}}$ to the classification of the molecule **M***_i_*. The conformer in set Σ making the highest contribution is selected as a bioactive conformer.

In order to validate the ability of MILES to identify the bioactive conformers, the contributions $f\left(C_{ij^{*}}\right)$ for the 12 seeded conformers, which were taken directly from co-crystallized complex structures, were calculated and ranked among all the conformers sampled for those 12 molecules.

### Classical QSAR methods without instance-based embedding

In order to examine the predictive performance of MILES, conventional classification approaches based on classical QSAR principles without instance-based embedding were tested for comparison. Since one molecule is defined as a bag of multiple conformers (instances), the pharmacophore fingerprint associated with a single molecule was obtained from the binary union of all of the pharmacophore fingerprints associated with the conformers of that molecule. The same occurrence-based filtering and significance analysis of pharmacophore fingerprints were performed to select the optimal subsets of the fingerprints which constituted the feature space for the classical QSAR studies. Three widely used classification algorithms including decision tree (DT) [20], 1-norm SVM [19], and random forest [21] were employed for comparison with MILES-SVM. The decision tree is a greedy method based on a recursive partitioning algorithm. The classification trees were constructed using the 'classregtree' function implemented in Matlab R2011b. The tree-based classification method can account well for multiple binding mechanisms [12]. Gini's diversity index was used for recursive partitioning, and the minimal number of molecules per tree leaf was set as 3 to terminate tree growing. The 1-norm SVM model is a statistical learning theory derived from the structural risk minimization principle and Vapnik-Chervonenkis (VC) dimension [22]. It is different from the tree-based method and served as an alternative comparison. Since the major drawback of DT is its low prediction caused by the overfitted tree-based structure, the ensemble learning method, random forests [21], can deliver improved prediction while retaining the appealing properties of tree-based methods. It is a collection of decision trees which are grown from bootstrapping samples of the original data without tree pruning, and has been demonstrated as one of the most powerful tools available for data exploration [23]. The Matlab implementation (randomforest-matlab v0.02) was used with default parameters.

## Results and discussion

### Data set preparation and division

According to the criteria used to label positive and negative molecules, the number of molecules in each of two classes was balanced for four data sets. Data set **I **has 266 molecules as positive and 258 molecules as negative; data set **II **has 253 molecules as positive and 284 molecules as negative; data set **III **has 307 molecules as positive and 188 molecules as negative; and data set **IV **has 122 molecules as positive and 128 molecules as negative. In terms of division of training and test sets, a stratified sampling was used to partition all four data sets into training and test sets at ratios around 3:1, respectively (Table 2).

**Table 2:** Data set statistics

Data set	No. of molecules in training set		No. of molecules in test set		Total no. of molecules
		
	Positive	Negative	Positive	Negative	
**I**	199	188	67	70	524
**II**	191	210	62	74	537
**III**	247	131	60	57	495
**IV**	94	93	28	35	250

### Conformational sampling

The molecules in different data sets had various conformational flexibilities, so the average number of conformers for each molecule was distinct for the four data sets (Table 3). The average number of conformers for each molecule was 43 in data set **I**, 89 in data set **II**, 86 in data set **III**, and 211 in data set **IV**. So the molecules in data set **IV **had the highest conformational flexibility. The feature space constructed through instance-based embedding only consisted of the instances from training bags, in other words, the conformers from the molecules in the training set. The molecules in the test set were not used in the construction of the instance-based feature space. So *n*_training _in Table 3 indicates the number of instance-based features used for embedding.

**Table 3:** Conformational sampling and pharmacophore fingerprints

Data set	*n*_training_ *^a^*	*n*_test_ *^b^*	*m*_pre-filtering_ *^c^*	*m*_post-filtering_ *^d^*	*m*_significant_ *^e^*	Δ^*f^
**I**	17249	5399	1872521	243721	2979	1.77
**II**	35434	12333	1670985	155220	14002	5.40
**III**	32528	9942	1636254	145996	1542	1.80
**IV**	41960	10746	13687602	161018	3467	1.66

*^a ^*The number of conformers in the training set; *^b ^*the number of conformers in the test set; *^c ^*the number of pharmacophore bits in the fingerprint originally enumerated; *^d ^*the number of pharmacophore bits in the fingerprint after filtering; *^e ^*the number of bits in the optimal subset of the pharmacophore fingerprint; *^f ^*the optimal threshold value to select truly significant pharmacophore bits.

### Significance analysis of pharmacophore models

Millions of pharmacophore models were originally enumerated for each data set, and the largest number of pharmacophore models was generated for data set **IV**. This correlated with the observation that the molecules in data set **IV **have the highest conformational flexibility. After occurrence-based filtering, only a small portion of the pharmacophore models was retained for each data set. For instance, 13% was retained for data set **I**, 9% for both data sets **II **and **III**, and 1% for data set **IV **(Table 3).

Significance analysis was subsequently performed upon those retained pharmacophore models. The threshold values were set to 100 equally spaced intervals from 0 to the largest difference between the ranking scores and reference scores. As the threshold value increases in a bottom-up manner, the number of falsely significant pharmacophore models decreases, and the number of truly significant models remains roughly constant. So the optimal threshold values (Δ*) for each data set can be obtained when the number of falsely significant pharmacophore models drops to zero (Table 3). Subsequently, the optimal subsets of the pharmacophore fingerprint bits were obtained for four data sets (Table 3). Only a very small portion of the fingerprint bits were significant for classification, namely 1% in data set **I**, 9% in data set **II**, 1% in data set **III**, and 2% in data set **IV**.

In the context of MIL, the optimal subsets of the binary strings were used to calculate the dissimilarity between two conformers for instance-based feature mapping. For the classical QSAR methods, the optimal subsets of the fingerprints were used as the 3D descriptors in the pharmacophore-based feature space for building classification models.

### Predictive performance of MILES and classical QSAR methods

In the MILES model, the only tuning parameter λ was determined by a grid search. Five replications of 5-fold cross-validation were performed to assess the classification accuracies at each point over a fixed grid which ranged from 2^-8 ^to 2^5 ^with exponential increment in base 2. The median values for the 5 replications were used to find the optimal tuning parameters. During the cross-validation, the instance-based feature space was dynamically defined, which means that the conformers from the molecules in the internal test set, after random split of the training set, were excluded from the feature space. As a result, the optimal tuning parameters as well as the number of prototype conformers were obtained for four dissimilarity measures (Table 4).

**Table 4:** Optimization of tuning parameter λ for MILES

Data set	Dissimilarity measure	Cross-validation*^a^*	λ	*n^b^*
**I**	Soergel	0.777	8.000	196
	Dice	0.761	4.400	165
	Manhattan*^c^*	0.803	4.400	130
	Rogers-Tanimoto	0.801	4.000	153

**II**	Soergel	0.865	0.001	103
	Dice	0.865	0.001	85
	Manhattan*^c^*	0.877	0.022	63
	Rogers-Tanimoto	0.868	0.069	72

**III**	Soergel	0.899	0.001	94
	Dice	0.901	0.001	75
	Manhattan	0.934	0.550	63
	Rogers-Tanimoto*^c^*	0.935	4.400	46

**IV**	Soergel	0.579	0.003	125
	Dice	0.544	0.031	111
	Manhattan	0.690	0.550	87
	Rogers-Tanimoto*^c^*	0.689	6.800	78

*^a ^*The median classification accuracy for 5 replications of 5-fold cross-validation; *^b ^*the number of prototype conformers selected in the set Γ; *^c ^*The model selected based on the number of prototype conformers.

Based on the internal validation, the classification accuracies were similar within each data set using four different dissimilarity measures. However, the numbers of prototype conformers selected were much different. For instance, in data set **I **and **II**, Manhattan distance yielded the smallest subset of selected prototype conformers, but in data set **III **and **IV**, Rogers-Tanimoto yielded the smallest subset. Furthermore, Soergel distance yielded the largest subset for all the four data sets. The dissimilarity measure which yielded the smallest number of selected prototype conformers was chosen as the best MILES model and used later for comparison with classical QSAR models without instance-based embedding.

After finding the optimal λ, a MILES model was identified from the training set and applied to the test set. In addition to comparing classification accuracy, denoted as the proportion of correct predictions, Matthews Correlation Coefficient (MCC) [24] was also employed as a complementary indicator for the predictive performance. MCC is defined as:

(7) $$MCC=\frac{TP\times TN-FP\times FN}{\sqrt{\left(TP+FP\right)\left(TP+FN\right)\left(TN+FP\right)\left(TN+FN\right)}}$$

where TP is true positive, TN is true negative, FP is false positive, and FN is false negative. MCC not only takes into account true positives and true negatives as classification accuracy does, but also false positives and false negatives. Thus it is considered as a balanced measure of the performance of binary classification (Table 5).

**Table 5:** Predictive performance for different dissimilarity measures

Data set	Dissimilarity measure	Training set		Test set	
		
		Accuracy	MCC	Accuracy	MCC
**I**	Soergel	0.972	0.944	0.854	0.714
	Dice	0.979	0.959	0.825	0.653
	Manhattan*^a^*	0.941	0.881	0.861	0.725
	Rogers-Tanimoto	0.961	0.923	0.861	0.725

**II**	Soergel	0.965	0.933	0.860	0.725
	Dice	0.965	0.933	0.868	0.745
	Manhattan*^a^*	0.978	0.956	0.904	0.807
	Rogers-Tanimoto	0.973	0.946	0.897	0.793

**III**	Soergel	0.989	0.977	0.846	0.706
	Dice	0.989	0.977	0.855	0.717
	Manhattan	0.979	0.954	0.838	0.686
	Rogers-Tanimoto*^a^*	0.947	0.885	0.846	0.711

**IV**	Soergel	0.904	0.823	0.667	0.301
	Dice	0.904	0.823	0.635	0.307
	Manhattan	0.957	0.918	0.714	0.433
	Rogers-Tanimoto*^a^*	0.898	0.811	0.794	0.584

*^a ^*The model selected based on the number of prototype conformers.

In accordance to classification accuracy and MCC, the performance of different dissimilarity measures was dataset-specific. For data set **I**, both the Manhattan and Rogers-Tanimoto distances were top-ranked and performed equally well on the test set, whereas on the training set, the Soergel and Dice distances performed much better than the Rogers-Tanimoto and Manhattan distances, and the Rogers-Tanimoto distance performed slightly better than the Manhattan distance. In addition, the results did not change after removing the 12 seeded conformers which were used for the validation of identifying bioactive conformers. For data set **II**, the Manhattan distance was top-ranked on both training set and test set. For data set **III**, the Dice distance was top-ranked on both training and test sets. For data set **IV**, the Rogers-Tanimoto distance performed much better on the test set, but on the training set it was not the top-ranked dissimilarity measure. It is interesting that for data sets **I, II**, and **III **the differences in the predictive performances of the four dissimilarity measures were very small, whereas for data set **IV **the differences were much larger. This may be caused by the high structural diversity in data set **IV**. The small difference in dissimilarity measures had a big impact on the predictive performance. The other interesting observation was that the classification accuracy and MCC provided the same indications for the predictive performance, which means that the data sets in the present work were highly balanced and good for benchmark studies.

After comparing the predictive performance of different dissimilarity measures in the MILES model, the predictive performance of MILES models was compared with that of conventional classification approaches, which are based on classical QSAR principles without instance-based embedding. To find the optimal λ for 1-norm SVM on the basis of classical QSAR principles, the same procedure was employed, which resulted in the minimal subset of the most important pharmacophore models (Table 6).

**Table 6:** Optimization of tuning parameter λ for 1-norm SVM

Data set	Cross-validation*^a^*	λ	*n^b^*
**I**	0.693	0.001	223
**II**	0.880	2.000	80
**III**	0.912	0.016	77
**IV**	0.598	0.125	89

*^a ^*The median classification accuracy for 5 replications of 5-fold cross-validation; *^b ^*the number of important pharmacophore bits.

For data set **I**, the 1-norm SVM without instance-based embedding overfit the training set, producing perfect prediction on the training set and poor prediction on the test set. However, MILES performed fairly well on both the training and test sets without overfitting. MILES performed much better than decision trees and slightly worse than random forests in terms of the predictive power on the test set. For data set **II**, MILES was highly competitive with the other classical QSAR methods, yielding the second best prediction on both training and test sets, while 1-norm SVM without embedding provided the best prediction on the training set but suffered from overfitting and decision trees produced the best prediction on the test set. For data set **III**, MILES performed slightly worse than random forests, but better than the other two methods, based on the predictions on the test set. Although MILES using Dice distance was not selected, since it yielded a large number of selected prototype conformers, it performed equally as well as random forests on the test set. For data set **IV**, MILES significantly outperformed the other approaches based on the predictions on the test set. For all the data sets, 1-norm SVM without embedding overfit the training set, yielding the best predictions on the training sets and relatively low predictions on the test sets. However, after instance-based embedding, MILES performed fairly well on both training and test sets without overfitting, and its predictive power was highly comparable with other conventional QSAR approaches (Table 7). It was interesting that the classification accuracy and MCC provided the same indications again, even for the comparison of different QSAR approaches.

**Table 7:** Predictive performance for different models

Data set	Methods	Training set		Test set	
		
		Accuracy	MCC	Accuracy	MCC
**I**	MILES*^a^*	0.941	0.881	0.861	0.725
	Decision tree	0.915	0.830	0.781	0.569
	1-norm SVM	1.000	1.000	0.832	0.668
	Random forest	0.995	0.990	0.891	0.783

**II**	MILES*^a^*	0.978	0.956	0.904	0.807
	Decision tree	0.955	0.913	0.919	0.837
	1-norm SVM	0.980	0.961	0.882	0.765
	Random forest	0.945	0.896	0.868	0.754

**III**	MILES*^b^*	0.947	0.885	0.846	0.711
	Decision tree	0.966	0.924	0.838	0.682
	1-norm SVM	0.995	0.988	0.812	0.624
	Random forest	0.982	0.959	0.855	0.717

**IV**	MILES*^b^*	0.898	0.811	0.794	0.584
	Decision tree	0.914	0.829	0.698	0.398
	1-norm SVM	0.952	0.906	0.714	0.418
	Random forest	0.936	0.877	0.698	0.392

*^a ^*Manhattan dissimilarity measure; *^b ^*Rogers-Tanimoto dissimilarity measure.

### Identification of bioactive conformers

After examining the predictive ability of MILES, we tested the ability of MILES in the pursuit of the bioactive conformers. Due to the lack of experimental data, the validation can only be made for the molecules in data set **I**. We made use of 12 co-crystallized structures of GSK-3 with bound small molecules, which adopt bioactive conformers in the complex structures (Table 8). See additional file 1 for the chemical structures of 12 small molecules. The direct comparison between the structures of the co-crystallized conformers and the ones from conformational sampling is difficult and sometimes impossible, since the conformational sampling plus structural minimization may not provide the exact same conformations found in the co-crystallized complex, due to the lack of protein environment in the conformational search process. So we adopted an indirect validation method. We seeded the 12 co-crystallized conformers in the set of sampled conformers generated through extensive exploration of conformational space. Then we calculated their contributions $f\left(C_{ij^{*}}\right)$ to the classification of the relevant positive molecules as described above (Table 8).

**Table 8:** Validations on the prediction of bioactive conformers

ID*^a^*	Name*^b^*	PDB ID*^c^*	Contribution*^d^*	Rank*^e^*	*n*_*i*_^*f*^
23	AR	1Q5K	2.792	3	117
37	Benzoimidazole-1	2O5K	0	N.A.*^g^*	138
50	Jonjon-1	2OW3	2.827	6	38
59	LM-4	1Q3W	0.858	1	2
60	LM-5	1UV5	11.941	1	3
77	LM-29	1Q41	8.576	2	7
97	Maleimide	1R0E	0	N.A. *^g^*	121
98	OxaD-0	3F7Z	10.629	1	53
99	OxaD-00	3GB2	4.637	2	9
153	Pyzo-11	3L1S	10.371	1	11
198	RM-0	1Q4L	5.568	2	25
199	Staurosporine	1Q3D	22.359	1	5

*^a ^*Molecule index in the data set; *^b ^*molecular name in the data set; *^c ^*Protein Data Bank index for the protein structure from which the experimental conformer was extracted; *^d ^*contribution $f\left(C_{ij^{*}}\right)$ calculated using equation 6; *^e ^*the rank in the set of contributions; *^f ^*the number of conformers for each molecule; *^g ^*the rank cannot be determined and the conformer was predicted to be irrelevant to classification based on the MILES method.

Three out of 12 molecules are highly flexible, adopting more than 100 conformers. For these three, MILES only correctly predicted one co-crystallized conformer as the third most significant conformer contributing to the classification of the molecule named AR. It incorrectly predicted the other two co-crystallized conformers as irrelevant conformers in terms of the contribution to the classification of benzoimidazole-1 and maleimide.

But for the molecules adopting less than 100 conformers, which had relatively rigid structures, MILES correctly predicted all the co-crystallized conformers as significant conformers for the classification of positive molecules. Five co-crystallized conformers were predicted to be the most significant conformers, i.e., the bioactive conformers; three co-crystallized conformers were predicted to be the second most significant conformers; and one co-crystallized conformer was predicted to be the sixth most significant conformer, based on the calculations of $f\left(C_{ij^{*}}\right)$. So the pursuit of bioactive conformers is easy for relatively rigid molecules and relatively more difficult for the highly flexible ones.

## Conclusions

We have successfully implemented a multiple-instance learning (MIL) framework, multiple-instance learning via embedded instance selection (MILES), for drug activity prediction. The molecules and relevant conformers were described using superior 3D descriptors, pharmacophore fingerprints, encoded as binary strings. The instance-based embedding was accomplished using dissimilarity measures designed for calculations on binary strings. The joint feature selection and classification was accomplished using a wrapper model based on 1-norm SVM. We have used the approach for the prediction of the labels of molecules interacting with four therapeutic targets, including GSK-3, CBrs, and P-gp. Based on the predictive performance, our proposed approach was highly competitive with conventional classification approaches based on classical QSAR principle. However, the proposed method, unlike conventional classification approaches, can also predict the contributions of individual conformers for each molecule and further can identify the putative bioactive conformer. These unique characteristics make the proposed method very useful for the pursuit of biologically significant conformers. Finally, we have validated that the proposed approach is highly useful in the pursuit of bioactive conformers.

## List of abbreviations used

MIL: multiple-instance learning; MILES: multiple-instance learning via embedded instance selection; SVM: support vector machine; QSAR: quantitative structure-activity relationship; GSK-3: glycogen synthase kinase-3; GPCR: G protein-coupled receptor; P-gp: P-glycoprotein; SOM: self-organizing map; MCC: Mathews correlation coefficient.

## Competing interests

The authors declare that they have no competing interests.

## Authors' contributions

GF, YC, DEW, and RJD planned the research. GF prepared the GSK3 and P-gp data sets. HL, RYP and PRD prepared the CB data sets and used preliminary machine learning methods for classification of CB compounds. GF prepared the computer code and ran the calculations with the assistance of XN. GF, YC, DEW, and RJD analyzed the results. GF wrote the manuscript with assistance from YC and RJD. All authors carefully reviewed the manuscript before submission.

## Supplementary Material

Additional file 1 **This file contains the chemical structures of 12 co-crystallized molecules (from GSK-3 structures) in PDB database, associated with the ID number, names, and PDB ID**. Click here for file
