# Supplementary material for: Implementation of multiple-instance learning in drug activity prediction
Source: BMC Bioinformatics. 2012 Sep 11;13(Suppl 15):S3. doi: 10.1186/1471-2105-13-S15-S3 (PMC3439725; doi:10.1186/1471-2105-13-S15-S3)
Supplement: Additional file 1 — This file contains the chemical structures of 12 co-crystallized molecules (from GSK-3 structures) in PDB database, associated with the ID number, names, and PDB ID. [file 1471-2105-13-S15-S3-S1.pdf]

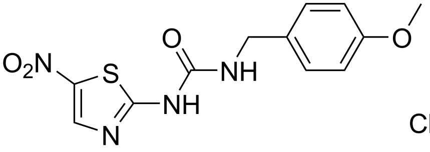

ID: 23
Name: AR
PDB ID: 1Q5K

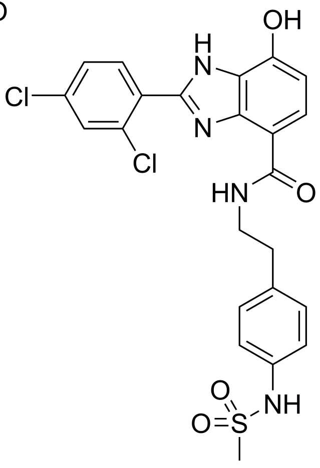

ID: 37
Name: Benzoimidazole-1
PDB ID: 2O5K

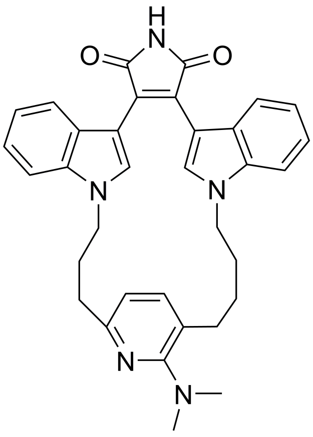

ID: 50
Name: Jonjon-1
PDB ID: 2OW3

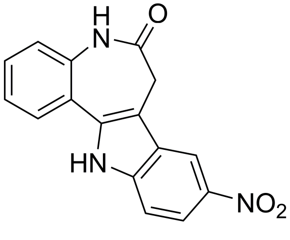

ID: 59
Name: LM-4
PDB ID: 1Q3W

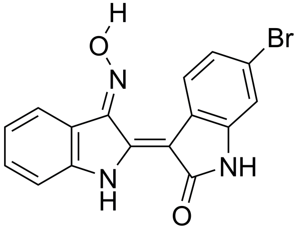

ID: 60
Name: LM-5
PDB ID: 1UV5

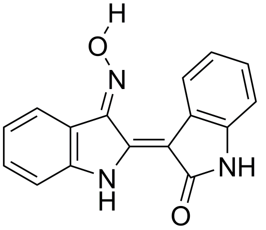

ID: 77
Name: LM-29
PDB ID: 1Q41

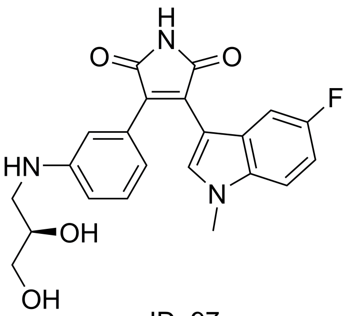

ID: 97
Name: Maleimide
PDB ID: 1R0E

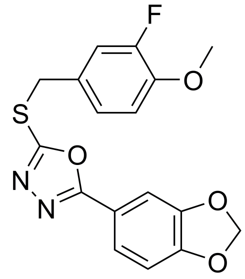

ID: 98
Name: OxaD-0
PDB ID: 3F7Z

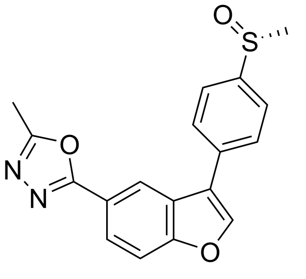

ID: 99
Name: OxaD-00
PDB ID: 3GB2

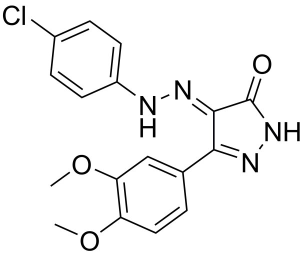

ID: 153
Name: Pyzo-11
PDB ID: 3L1S

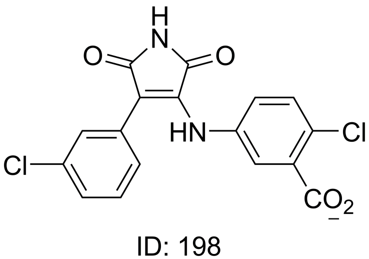

ID: 198
Name: RM-0
PDB ID: 1Q4L

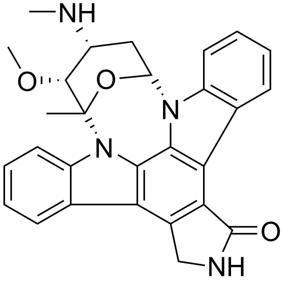

ID: 199
Name: Staurosporine
PDB ID: 1Q3D
